# Using Satellite Remote Sensing to Estimate Rangeland Carrying Capacity for Sustainable Management of the Marismeño Horse in Doñana National Park, Spain

**DOI:** 10.3390/ani15243507

**Published:** 2025-12-05

**Authors:** Emilio Ramírez-Juidias, Ángel Díaz de la Serna-Moreno, Manuel Delgado-Pertíñez

**Affiliations:** 1 Instituto Universitario de Arquitectura y Ciencias de la Construcción (IUACC), Universidad de Sevilla, 2 Reina Mercedes Avenue, 41012 Seville, Spain; erjuidias@us.es; 2 Departamento de Agronomía, Escuela Técnica Superior de Ingeniería Agronómica, Universidad de Sevilla, Ctra. Utrera Km 1, 41013 Sevilla, Spain; adiazdelaserna97@gmail.com

**Keywords:** satellite imageries, utilization of grazing resources, *Doñana* horses, South of Iberian Peninsula, Mediterranean wetlands

## Abstract

Rangeland degradation poses a serious challenge for the sustainable management of free-ranging livestock in Mediterranean wetlands. In *Doñana* National Park, Spain, the endangered *Marismeño* horse depends exclusively on natural forage, making it essential to monitor vegetation productivity and grazing suitability under increasing climate variability. This study presents a satellite-based assessment of rangeland carrying capacity to support the adaptive management of this iconic breed. A six-year time series (2015–2020) of 1242 images from Landsat 8 OLI/TIRS and Sentinel-2 (L1C/L2A) was processed using ILWIS and Python-based workflows to derive vegetation indices (GNDVI, NDMI) and model aboveground biomass, forage energy, and grazing pressure across five grazing units. Results revealed strong seasonal cycles, with biomass and nutritive value peaking in spring and declining sharply in summer. Ecotonal zones such as *La Vera y Sotos* acted as crucial refuges during drought-induced resource shortages. The harmonized multi-sensor approach demonstrated high reliability for mapping forage dynamics and assessing carrying capacity at fine scales. This remote sensing framework offers an effective, scalable tool for sustainable livestock management in *Doñana*, directly supporting biodiversity conservation and the long-term resilience of Mediterranean rangeland ecosystems.

## Simple Summary

The *Marismeño* horse, a free-ranging and endangered livestock breed native to *Doñana* National Park in southern Spain, depends entirely on natural pastures within a highly dynamic wetland ecosystem. Seasonal drought, flooding, and salinity changes strongly affect the availability and nutritional quality of its forage, yet reliable tools to monitor these conditions have been limited. This study aimed to develop a satellite-based method to estimate vegetation growth, forage quality, and grazing pressure across *Doñana*’s main grazing units over a six-year period (2015–2020). By analyzing more than one thousand satellite images from Landsat 8 and Sentinel-2 and integrating them with field data, we identified clear seasonal patterns, with spring showing the highest forage production and late summer the lowest quality. Ecotonal zones such as *La Vera y Sotos* acted as critical refuges for grazing when the marshes dried out. The results demonstrate that remote sensing can provide managers with timely and objective information to guide adaptive herd management, safeguard the ecological integrity of the park, and help preserve this emblematic horse breed. This approach can also be applied to other Mediterranean and semi-arid ecosystems facing similar challenges from climate variability and water scarcity.

## 1. Introduction

Doñana National Park, located in Andalucía, southwestern Spain, is one of the most valuable and diverse ecosystems in Europe. This protected area is home to a wide variety of habitats, including marshes, dunes, forests, and grasslands, which support a rich biodiversity and provide essential ecosystem services [1,2]. The native vegetation has historically coexisted with large herbivores, including both wild and domesticated species. The park’s provisioning services include livestock, primarily consisting of three native breeds: the *Marismeño* horse, the *Mostrenca* cow, and the *Andalusian churra* sheep [2]. Nowadays, its economic significance has given way to its role as a cultural icon of the region [2]. The event known as the “*Saca de las Yeguas* (the Mares’ Roundup)” in particular reflects the deep connection between the local community and this horse breed, making it a central element of local identity and the intangible heritage of Andalusia [2,3].

The *Marismeño* horse is included in the Official Catalogue of Livestock Breeds of Spain [4], but due to its low census, it is also classified as an endangered breed [5]. Among its particularities are its rusticity and adaptation to the marsh environment, feeding exclusively on natural grazing resources, without receiving any nutritional supplementation [6]. For the conservation of this breed, as a heritage of livestock biodiversity and the sociocultural traditions that surround it, sustainable resource management is key to preserving the ecological balance of *Doñana* Park.

The sustainable management of this special ecosystem, where the direct interaction of livestock species such as the *Marismeño* horse with natural resources, especially grasslands, without human intervention, implies the need to estimate the carrying capacity to avoid habitat degradation. Overstocking can lead to soil compaction, nutrients and vegetation loss, and hydrological alterations [7], affecting not only the local fauna and flora, but also the heritage value of the *Doñana* park as a Biosphere Reserve. Among the factors influencing carrying capacity [8], “decision factors” that a farmer or manager must establish (for instance, by relocating, confining, or supplementally feeding livestock) would currently be less influential, due to the free-range grazing system and the difficulty of real-time decision-making. Instead, those that are inherently and not likely to be changed, such as soil type, topography, and climate, are more influential. Indeed, the simultaneous occurrence of water scarcity and high temperatures in summer, defining features of Mediterranean environments, likely constitutes one of the key factors affecting ecosystem dynamics in most *Doñana*’s natural areas [1].

The assessment of the carrying capacity requires high-resolution monitoring and detailed knowledge of numerous ecological parameters, supported by a robust database that includes spatial vegetation patterns, forage quality metrics (specifically the energetic nutritive value of distinct plant communities) biomass availability, the spatial and temporal dynamics of grazing zones, as well as data on animal census and social structures [9]. The determination of carrying capacity entails a highly complex and inherently stochastic process, which explains its absence in numerous regions where adequate data and methodological frameworks are lacking [10]. Recent research integrating satellite-derived datasets with grazing utilization indicators suggest that remote sensing methodologies, when complemented by field-based data, constitute effective tools for rangeland monitoring and sustainable management [10,11,12]. Some previous studies in *Doñana* have used remote sensing data and techniques to investigate the primary production among vegetation types as well as the response to factors such as climate change and grazing pressure [13,14]. However, further work is required to support the development of long-term management strategies grounded in plant production and consumption scenarios, as well as to create near real-time tools that enable swift adjustment of herbivore stocking rates in response to vegetation output [14].

In this context, satellite remote sensing has become a useful way to keep an eye on the state of rangeland. It can give you spatially explicit, repeatable, and long-term datasets at different scales [15]. According to [16], multispectral and hyperspectral imagery can be used to measure the health of plants, the amount of biomass available, and the quality of forage over large, diverse areas like *Doñana*. Remote sensing uses the spectral signatures of plants to create vegetation indices, which are good indicators of photosynthetic activity, canopy structure, and nutrient status. These indices, when used with ecological models, let us measure important things like the amount of biomass above ground, the nutritional value of pastures, and how grazing resources change with the seasons [17].

In accordance with [18], using remote sensing to monitor rangeland has many benefits over traditional field-based methods as well. Field measurements are necessary for calibration and validation, but they usually do not cover a lot of space or time, which means they need a lot of work and resources. Satellite images, on the other hand, give us continuous observations over time, which lets us look at long-term trends, find sudden changes, and measure interannual variability caused by climate changes [19]. This ability is especially useful in Mediterranean ecosystems, where the strong seasonal changes in temperature and rainfall cause vegetation growth and forage availability to change quickly.

For the *Marismeño* horse, which only eats natural forage and does not receive any extra food, remote sensing is a unique way to connect vegetation dynamics with animal census data and grazing pressure [20]. According to [20], it is possible to estimate the carrying capacity of different grazing areas in *Doñana* by using satellite-derived indicators to model the availability of biomass and its nutritional value. This, in turn, helps create management plans that protect both the marshlands’ ecological health and the survival of the endangered horse breed. Additionally, the capacity to observe vegetation responses to grazing intensity offers understanding into feedback mechanisms that influence habitat resilience and the long-term functioning of ecosystems [21].

Recent improvements in remote sensing platforms and data processing have made operational monitoring even more possible [22,23]. Landsat 8 and Sentinel-2 are examples of medium-resolution sensors that can show the different types of habitats in *Doñana* and how they change over time. As cloud-based platforms and data mining techniques become easier to use, it becomes easier to work with large datasets [23]. This cuts down on the time between obtaining an image and analyzing it. At the same time, machine learning and predictive modeling techniques make it possible to create algorithms that turn spectral data into biophysical variables that are directly related to the productivity and nutritional quality of rangelands [24].

In protected areas like *Doñana*, where conservation goals must be balanced with the sustainable use of natural resources, it is especially important to use these new technologies in rangeland management. As is well known, and according to [25], using remote sensing to make predictive models of forage production and quality helps with short-term decisions about stocking rates and long-term plans to protect biodiversity and cultural heritage. Remote sensing is a key part of adaptive management because it helps protect the ecological, cultural [26], and economic values of the *Marismeño* horse for future generations.

In another vein, it is very important to take into account that various methodologies have been developed to evaluate grazing potential and carrying capacity through remote sensing techniques, offering valuable support for evidence-based ecological management strategies [27]. Recent research [28] has utilized the MODIS Net Primary Production (NPP) dataset to evaluate ecosystem productivity in relation to livestock densities, facilitating long-term monitoring of biomass accumulation and interannual variability at regional to continental scales. These methods have given us important starting points for figuring out how primary production is spread out over space in different climates and management styles. But because MODIS has a coarse spatial resolution of 250–1000 m, it is not very useful for landscapes like *Doñana* that are very different from each other. In these areas, small-scale hydrological and topographic gradients control vegetation patterns and the availability of forage.

More research has looked into finer-scale multispectral and hyperspectral datasets, especially from the Sentinel-2 and Landsat 8 missions. These datasets have better spatial and temporal resolution, which makes them better for monitoring rangelands. Sentinel-2 images have a resolution of 10 to 20 m and are sensitive to the red-edge and shortwave infrared bands. This makes it possible to create vegetation indices that are sensitive to both biomass and water content, like the Green Normalized Difference Vegetation Index (GNDVI) and the Normalized Difference Moisture Index (NDMI) [29]. These indices have been effectively utilized to measure the spatial variability of forage productivity, to differentiate between halophytic and mesophytic plant communities, and to track seasonal variations in marsh and grassland ecosystems [29]. Landsat 8 data, collected by the Operational Land Imager (OLI) and Thermal Infrared Sensor (TIRS), have also been widely used to estimate biophysical parameters like Leaf Area Index (LAI), Fraction of Absorbed Photosynthetically Active Radiation (fAPAR), and Land Surface Temperature (LST) [30]. These are important indicators for measuring plant stress, water availability, and the overall health of rangeland [31].

Beyond these optical systems, radar and LiDAR-based methodologies [31,32] have gained attention for their ability to penetrate clouds and provide information on vegetation structure and canopy height, parameters directly linked to standing biomass and grazing suitability. For instance, Sentinel-1’s Synthetic Aperture Radar (SAR) data has been used to find changes in the amount of water that floods marshes over time and to map how easy it is for animals to graze when the water level changes. LiDAR-derived elevation models have been combined with optical images to better show microtopographic features that affect the distribution of pastures and the movement of large herbivores.

Recent methodological advancements have concentrated on the amalgamation of multisource data via machine learning and deep learning techniques, which improve prediction accuracy by elucidating nonlinear correlations between spectral indices and field-measured biomass. For example, Convolutional Neural Networks (CNNs) and Random Forest algorithms [33] have shown that they can accurately model aboveground biomass from Sentinel and Landsat images, even when the vegetation structure is complicated and there are different types of land cover. These changes have made it possible to make dynamic, spatially explicit models of carrying capacity that can be updated all the time as new images come in.

Previous studies have demonstrated the potential of remote sensing for assessing grazing resources and carrying capacity, mainly through coarse-resolution datasets such as MODIS-derived Net Primary Production (NPP) or NDVI time series [28]. However, these approaches often lack the spatial detail necessary to capture the fine-scale heterogeneity of Mediterranean wetlands like *Doñana*, where hydrological gradients and vegetation mosaics determine forage availability [29,31]. In this study, we advance these methodologies by integrating multi-sensor satellite imagery (Sentinel-2 and Landsat 8) with vegetation indices sensitive to both chlorophyll activity and moisture content, allowing a more accurate and dynamic estimation of forage biomass and nutritive value. This multi-sensor, high-resolution framework represents a novel application of Earth observation tools for the sustainable management of free-ranging livestock under fluctuating environmental conditions.

The motivation for this study arises from the need to reconcile livestock management with the conservation goals of *Doñana* National Park, where the *Marismeño* horse depends entirely on natural grazing resources. Despite the existence of numerous remote sensing studies addressing vegetation productivity in rangelands, few have focused on Mediterranean wetlands characterized by high hydrological variability and fine-scale spatial heterogeneity [9,10,11,12,13,14]. This research aims to fill that gap by developing a satellite-based methodology that combines spectral indicators of vegetation condition with ecological modeling to estimate forage availability, nutritive value, and carrying capacity. The resulting framework not only enhances scientific understanding of rangeland dynamics but also provides a practical decision support tool for sustainable management and conservation of free-ranging livestock in climate-sensitive wetland ecosystems.

In light of these challenges, this study aims to develop and validate a satellite remote sensing–based approach for estimating rangeland carrying capacity and forage dynamics to support the sustainable management of the *Marismeño* horse in *Doñana* National Park. The paper is structured as follows. Section 2 (Materials and Methods) describes the study area, the satellite datasets and processing workflow, and the analytical framework implemented in Python for calculating vegetation indices, biomass, and forage quality. Section 3 (Results) presents the spatial and temporal patterns of biomass production, nutritive value, and grazing pressure derived from multi-year satellite observations. Section 4 (Discussion) interprets these findings in the context of ecological processes, landscape heterogeneity, and conservation management, emphasizing the implications for adaptive strategies under climate variability. Finally, Section 5 (Conclusions) summarizes the main outcomes, methodological advances, and broader applications of this approach for the sustainable management of free-ranging livestock systems in Mediterranean wetlands and other climate-sensitive ecosystems.

## 2. Materials and Methods

### 2.1. Study Area

#### 2.1.1. Location and General Setting

**Figure 1:**
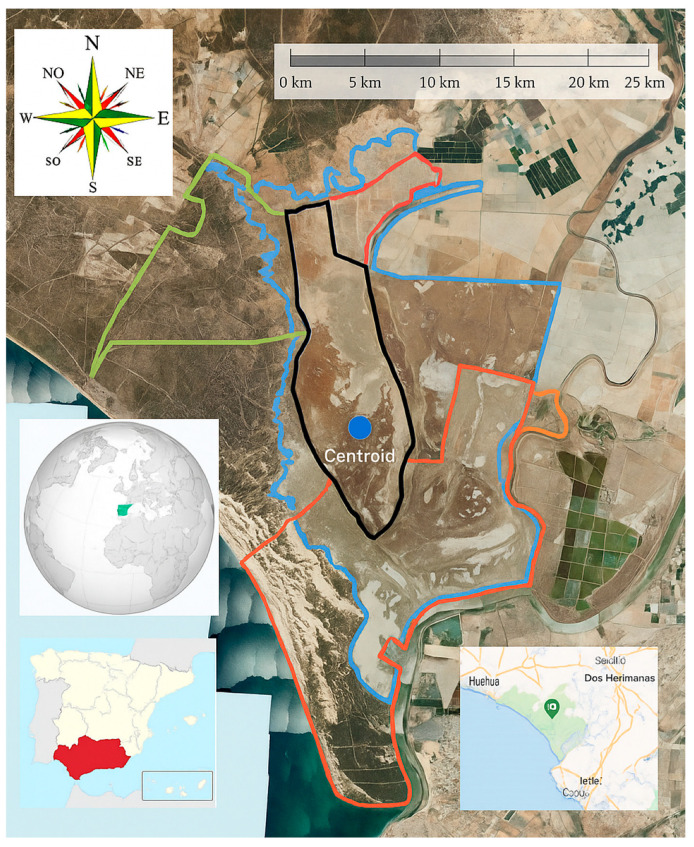
Location of the study area. Centroid UTM geographic coordinates under the ETRS-89 Datum, Zone 30 (Latitude: 37.0617° N; Longitude: 6.4960° W). (in blue the *Doñana* saltmarshes, in red *El Rincón*, in green *La Vera y Sotos*, in dark orange *Las Nuevas y Marismillas*, in light orange *Matochal* and in black *Marisma de Hinojos*).

The study (see Figure 1) was performed in five representative grazing units in *Doñana* National Park, southern Spain, where the *Marismeño* horse is customarily reared in free-range settings. These units were chosen because they are ecologically diverse and important for horse grazing. *El Rincón* is a mix of marshland and upland areas that serves as a safe haven during seasonal flooding and has a wide range of food sources. *La Vera y Sotos* is ecotonal area that lies between marsh and forest. It has sandy soils, seasonal flooding, and pastures that are mostly made up of *Juncus maritimus* and annual grasses. *Las Nuevas y Marismillas* is marshy area with a lot of water movement and changing salinity. The plants that grow there include both perennial grasses (*Cynodon dactylon*) and halophytic species that can grow in salty soil. *Matochal* is in the northern part of the park, which is mostly covered in seasonal flooding and a lot of herbaceous plants, most of which are ciperaceous communities. Last but not least, *Marisma de Hinojos* is one of the largest and most typical wetland areas. It has clay soils and halophytic plants like *Sarcocornia* and *Salicornia* spp. that are important for grazing at different times of the year.

The study area is located in southwestern Andalusia, Spain, within the *Doñana* National Park and the marshlands that surround it [34]. The protected area is made up of parts of the provinces of Huelva, Seville, and Cádiz. It is known for its high ecological value and the wide range of habitats it contains. The main national park and buffer zones are in the larger *Doñana* region, which is more than 1300 km^2^ in size.

In accordance with [34], the landscape is very flat, with elevations ranging from sea level to about 40 m above mean sea level. Large areas of the marshlands are flooded or have water tables that change with the seasons. A network of tidal channels (locally called *caños*) and freshwater streams drain the area. A spit and dune complex on the seaward side of the area separates the marshes from the Atlantic Ocean. The Guadalquivir River flows into this wetland-deltaic system, which affects the marsh’s sediment dynamics, salinity gradients, and hydrological regimes. Geomorphology shows how sediments built up during the Holocene, how the sea came in, and how old shallow lagoons slowly filled up.

#### 2.1.2. Climate, Hydrology, and Soils

*Doñana* has a Mediterranean climate, which means that winters are cool and wet, and summers are hot and dry, with strong seasonality. The average amount of rain is moderate, but it varies a lot from year to year. Most of the rain falls from late autumn to spring. In the summer, on the other hand, water is often scarce, and the rate of evaporation and transpiration is high, which can limit the growth of plants and the availability of food [34].

The marshes are dynamic in terms of hydrology: some areas are always flooded, others are flooded only during certain seasons, and some areas may dry out during dry spells. The levels of groundwater and surface inflows are two of the most important factors that affect how marshes flood. Changes in aquifer recharge, river flow patterns, or the removal of groundwater by people can have a big effect on the marsh’s hydrology, which in turn affects where plants grow and how much they grow [34].

Soils in marshes are usually hydric, with fine textures, high levels of organic matter in some areas, and salinity levels that change, especially in areas that are close to tidal influence or where brackish water comes in [35]. The soil type and waterlogging pattern affect the rooting depth, nutrient availability, and plant community composition, which in turn affect the potential for forage.

#### 2.1.3. Vegetation and Pasture Resources

The canopy in the *Doñana* marshes is very different from each other because of changes in water, salt, land, and disturbance [34]. Some important plant communities are emergent wetland species that grow in areas that are always or sometimes flooded, mixed *halophilous* marsh vegetation, and grassland or herbaceous communities that grow in drier areas or on the edges of wetlands. Because plants come in so many different shapes and sizes, the quality of forage (such as energy content, protein concentration, and fiber fractions) can change a lot over time and space [34].

In the past, native herbivores have grazed on these marsh pastures, and selective grazing pressure has probably changed the structure of the plant community. Because the *Marismeño* horse grazing is unmanaged and only depends on natural pastures [36], the availability, distribution, and nutritional quality of these plant communities are the most important things to think about when figuring out how much they can hold.

#### 2.1.4. Livestock Use, Land Tenure, and Management Constraints

The *Marismeño* horse grazes freely in the marshes and nearby land, without being fed or kept in a pen. The horses move around in marsh areas, picking spots based on how much food is available, how well the drainage works, and how easy it is to get to dry land. Land tenure is a mix of public protected areas and traditional communal or customary usage rights [37]. These rights are especially important for the *Saca de las Yeguas* (the annual mares’ roundup) and other cultural practices in the area.

There are big management problems in the area. Because the horses can roam freely, it’s hard for managers to move or add animals when there is not enough food for them [37]. Fencing or rotational grazing is also hard to do in this changing marsh landscape. Some parts of the marshes have historically practiced hydrological management, such as controlling inflows and drainage channels. However, there are limits to this because of conservation priorities, rules for protected lands, and the complicated relationships between hydrology, vegetation, and habitat integrity [34,37].

#### 2.1.5. Ecological and Conservation Context

*Doñana* is a UNESCO World Heritage Site and is also protected by Spanish and European conservation laws [38]. It is a biodiversity hotspot and is home to many endangered species from different groups. The marsh-grassland interface is very important for many birds, amphibians, and small mammals. This means that managing pastures for livestock must be balanced with larger environmental goals.

Climate change is causing droughts, changes in rainfall patterns, less aquifer recharge, groundwater extraction (especially for farming in nearby areas), and people changing the flow of rivers [34,39]. These pressures can cause the average marsh inundation periods to get shorter, the flooded habitat to get smaller [34], the soil salinity to rise, and the makeup of the plant community to change [39]. These kinds of changes could make it harder to find and use forage, which could affect the *Marismeño* horse’s long-term carrying capacity.

### 2.2. Satellite Data

Landviewer (EOS Data Analytics) (https://eos.com/landviewer, accessed on 2 December 2025) [40] provided satellite images for the time period from January 2015 to December 2020. In all, 1242 satellite images were processed. These included 136 Landsat 8 Collection 8 (LC8) scenes, 467 Sentinel-2 Level-2A (L2A) scenes (product with atmospheric correction), and 639 Sentinel-2 Level-1C (L1C) scenes (product without atmospheric correction). All of the images were taken in August 2025 and were in the Path 202 Row 34 “WRS-2” orbit. This acquisition strategy made sure that harmonized datasets were available for the whole study period. These datasets were then improved and pre-processed for analysis. It is important to highlight that the only reason for including both Sentinel-2 Level-2A (L2A) and Level-1C (L1C) datasets was to ensure a sufficiently large and statistically significant number of images to achieve fully reliable results (only cloud-free or low-cloud (<10%) images were used). By combining both pre- and post-atmospheric-correction products, we maximized temporal coverage and data availability across the six-year study period, without affecting the consistency of the analyses since all images were subsequently standardized and preprocessed following the same correction and harmonization procedures.

The harmonized dataset used in this study incorporated spectral bands from both Sentinel-2 (L1C and L2A) and Landsat 8 OLI sensors, specifically those required for computing the Green Normalized Difference Vegetation Index (GNDVI) and the Normalized Difference Moisture Index (NDMI). For the GNDVI, the green band (Sentinel-2 B3: 560 nm; Landsat 8 Band 3: 525–600 nm) and the near-infrared (NIR) band (Sentinel-2 B8: 842 nm; Landsat 8 Band 5: 845–885 nm) were used to evaluate photosynthetic activity and vegetation vigor. For the NDMI, the NIR band (Sentinel-2 B8; Landsat 8 Band 5) and the shortwave infrared (SWIR) band (Sentinel-2 B11: 1610 nm; Landsat 8 Band 6: 1560–1660 nm) were applied to assess canopy and soil moisture conditions. All reflectance values were radiometrically harmonized and resampled to a common spatial resolution of 30 m to ensure full compatibility between sensors before index computation.

### 2.3. Image Preprocessing

The Integrated Land and Water Information System (ILWIS) geographic information system was used for pre-processing. To improve the quality of the images, radiometric and atmospheric corrections were made [31,32,34]. In this regard, it is important to highlight that the radiometric corrections involved converting the original digital numbers (DN) of each image into Top-of-Atmosphere (TOA) reflectance values, followed by normalization across sensors to correct for differences in solar angle, acquisition geometry, and sensor calibration. This step ensured that pixel brightness values accurately represented surface reflectance rather than sensor or illumination artifacts, enabling direct comparison between Sentinel-2 and Landsat 8 datasets. On the other hand, the atmospheric corrections were then applied to remove the influence of aerosols, water vapor, and atmospheric scattering effects. For Sentinel-2 L2A imagery, atmospheric correction was performed using the Sen2Cor processor, which converts TOA reflectance to Bottom-of-Atmosphere (BOA) reflectance. For Landsat 8 OLI, the LEDAPS algorithm was employed to achieve an equivalent BOA correction. Both methods apply radiative transfer models to standardize surface reflectance values under varying atmospheric conditions. This dual correction process ensured that the harmonized imagery accurately reflected the biophysical properties of vegetation and surface moisture, thereby improving the precision of the computed vegetation indices (GNDVI and NDMI).

We made sure that all of the Sentinel-2 (L1C and L2A), Landsat 8 OLI, and TIRS datasets had the same spatial resolution (all imageries had a consistent spatial resolution of 30 m after resampled carried out prevented scale-related bias in vegetation index calculations and subsequent modeling) and were georeferenced to the same coordinate reference system. We used the cubic convolution algorithm to increase the size of the TIRS bands from 100 m to 30 m so that they would work with the OLI multispectral bands. After that, the nearest-neighbor algorithm was used to resample the data so that the original radiometric properties of the pixel brightness values were kept and spectral distortions were avoided [31,32].

After these changes were made, vegetation indices were calculated, such as the Green Normalized Difference Vegetation Index (GNDVI = (NIR − Green)/(NIR + Green)) and the Normalized Difference Moisture Index (NDMI = (NIR − SWIR)/(NIR + SWIR)), because they are sensitive to halophytic vegetation. This was done to measure both the biomass production (Dry Matter “DM”, r = 0.89; R^2^ = 0.79; *p* ≤ 0.001) and the nutritional quality (UFL = (UFL_Area based_/total area per plot in hectares), r = 0.923; R^2^ = 0.915; *p* ≤ 0.001) of the grazing resources (see Equations (1) and (2)).

Dry Matter (kg/ha) = −636.35 + 276.32 NDMI + 3798.18 GNDVI(1)

UFL_Area based_ = −4390.82 + 1906.61 NDMI + 26,207.442 GNDVI (2)

### 2.4. Data Processing and Analytical Workflow

**Figure 2:**
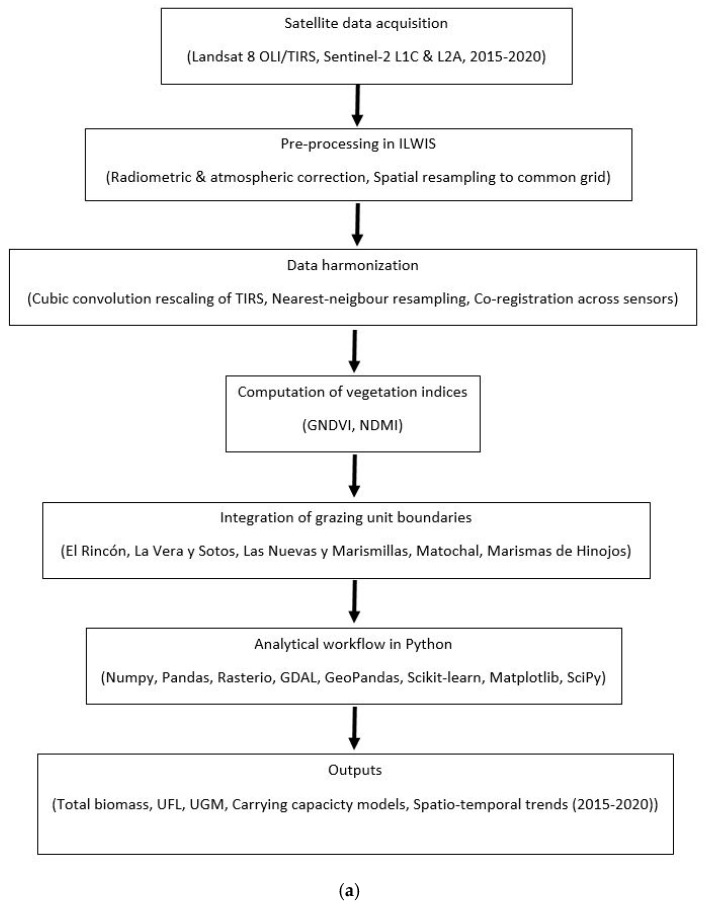

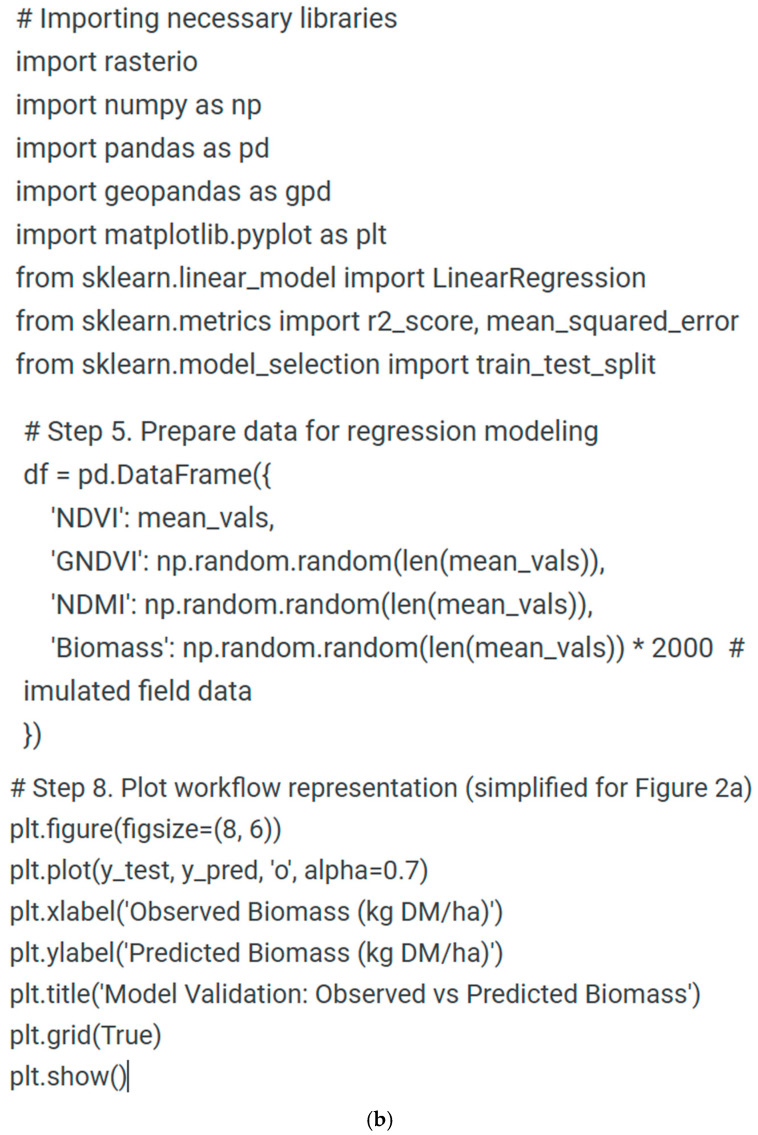
(**a**) Data processing workflow applied to estimate the rangeland carrying capacity for the *Marismeño* horse in *Doñana* National Park, Spain, during the period 2015–2020. (**b**) Python 3.9 summarized workflow for satellite data processing and modeling.

The analytical pipeline (Figure 2a) was built in Python 3.9, which made it easy to work with large image datasets and combine remote sensing data with ecological and animal census data. For this, a number of specialized libraries were used. NumPy 2.1.3 was used to do big math problems and handle multidimensional arrays, which made it easy to work with raster datasets. Pandas 2.2.2 make it easier to work with structured data by letting you combine spectral values, vegetation indices, and horse census data into single data frames that could be used for statistical modeling.

The summarized code below (Figure 2b) illustrates the complete workflow (from satellite image preprocessing to vegetation index calculation, regression-based biomass modeling, and validation) corresponding to the processes outlined in Figure 2a.

Rasterio 1.4 and the Geospatial Data Abstraction Library (GDAL) 3.10.0 were used to manage spatial data. These tools let you read, write, and geoprocess raster datasets in different formats, as well as do resampling, clipping, and mosaicking. GeoPandas 1.1.1 was used to work with vector data that showed the boundaries of farms and study sites. This made it possible to draw grazing units and do spatial overlay analyses. Matplotlib 3.10.7 and Seaborn 0.13.2 were used to make graphs, spectral profiles, and distribution maps of vegetation indices and modeled variables over time.

Scikit-learn 1.7.2 offered strong tools for regression analysis, machine learning algorithms, and cross-validation procedures for predictive modeling. These methods made it possible to find real-world links between vegetation indices and forage parameters like aboveground biomass (measured in kilograms of dry matter per hectare) and forage nutritive value (measured in forage units of lactation, UFL). SciPy 1.16.3 was also used for advanced statistical testing, curve fitting, and figuring out error metrics.

### 2.5. Field Data and Validation

The Spanish Association of *Marismeño* Horse Breeders (https://razamarismena.com/) [41] keeps yearly records of the number of animals, their demographics, and where they are on the study farms. This is how we obtained census data on *Marismeño* horse herds. We used these data along with estimates from satellites to check the accuracy of carrying capacity in livestock units (UGM = UFL/4.4, r = 0.91; R^2^ = 0.893; *p* ≤ 0.001) models and see how much grazing was going on. Field-based observations of pasture conditions, obtained from prior ecological studies and official management reports, served as benchmarks to corroborate remote sensing predictions of biomass availability and nutritional value.

### 2.6. Modeling Procedures

The modeling framework created predictive algorithms that linked satellite-derived vegetation indices to measured forage variables. We used 70% of the dataset to calibrate the regression analyses and the other 30% to test the models. The coefficient of determination (R^2^), root mean square error (RMSE), and mean absolute error (MAE) were used to measure how well the model worked. Spatial outputs were created as continuous surfaces showing the availability of biomass (DM) and its nutritional quality (UFL) for each study unit, grouped by farm boundaries. After that, these outputs were used to figure out the carrying capacity in livestock units (UGM) per hectare and to see how it changed over time from 2015 to 2020.

To derive Equations (1) and (2), we fitted ordinary least squares (OLS) linear regression models linking the satellite-derived vegetation indices (GNDVI and NDMI) with observed forage variables across the entire harmonized dataset (2015–2020). The model formulation followed the structure *Y* = *a* × *VI* + *b*, where *Y* is the estimated variable (biomass or forage energy), *VI* is the vegetation index value, and *a* and *b* are regression coefficients optimized during calibration. A 70/30 calibration–validation data split was applied to ensure that model performance was evaluated independently. The resulting regression equations thus reflect site-wide empirical relationships, enabling the generation of spatially explicit maps of biomass and forage energy across the grazing units in Doñana National Park.

## 3. Results

### 3.1. Climate Backdrop (2015–2020)

**Figure 3:**
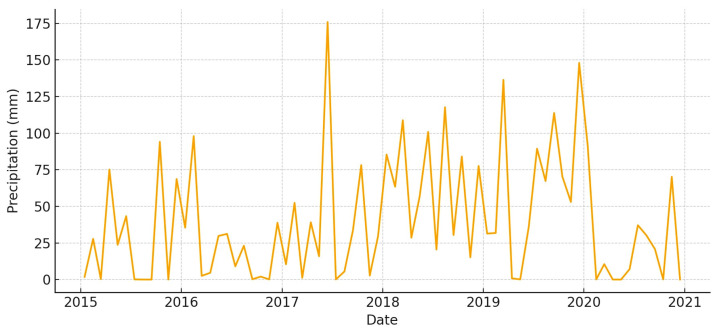
Monthly precipitation in *Doñana* (2015–2020).

**Figure 4:**
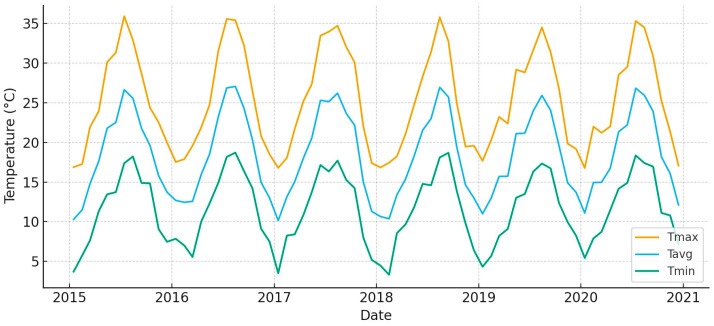
Monthly temperatures in *Doñana* (2015–2020).

According to [42] (https://en.climate-data.org/), the Mediterranean pulse can be seen in monthly precipitation, which changes a lot from year to year (Figure 3). From late fall to spring, wet-season totals build up, with occasional monthly peaks that are very high. Every year, the summer months are always dry, which creates a long period of water stress. Monthly temperatures follow a tight seasonal cycle (Figure 4), with a maximum temperature (T_max_) frequently exceeding 30 °C in summer and a minimum temperature (T_min_) falling below 10 °C in winter. This hydroclimatic background sets the stage for the timing of forage availability and the growth of plants across the marsh–vera gradient [34,35].

### 3.2. Multi-Year Vegetation and Forage Dynamics by Grazing Unit

#### 3.2.1. El Rincón

**Figure 5:**
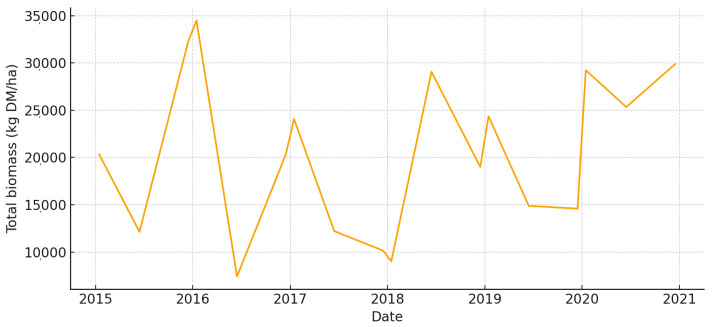
Total aboveground biomass (kg DM/ha) in *El Rincón* (2015–2020).

**Figure 6:**
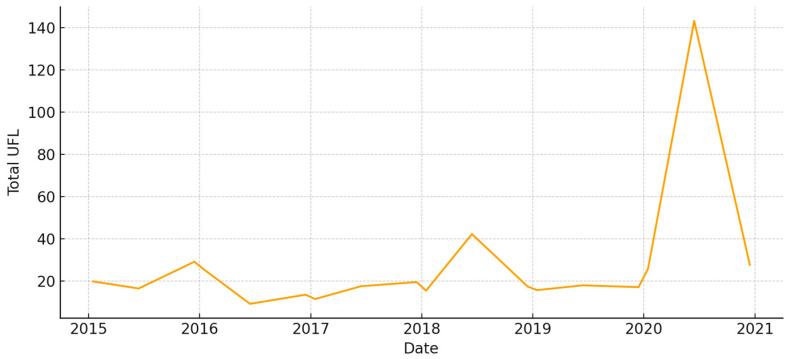
Total forage energy (UFL) in *El Rincón* (2015–2020).

**Figure 7:**
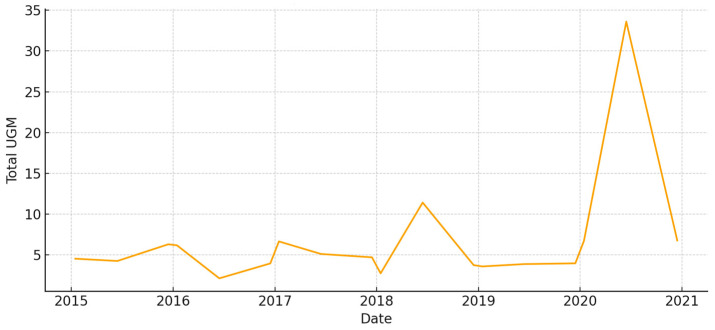
Total grazing pressure (UGM) in *El Rincón* (2015–2020).

The total aboveground biomass (kg DM ha^−1^) shows a clear seasonal pattern, with more of it building up during the cool, wet months and then dropping off sharply in the summer (Figure 5). Occasional spikes in total biomass correspond to favorable winter–spring rainfall. Total UFL follows biomass during green-up but drops more quickly during summer senescence (Figure 6). This shows that quality and quantity are not linked in the late season. Total UGM has short lags compared to resource pulses (Figure 7), which means that grazing pressure moves toward patches that are easier to reach and of higher quality as low-marsh pastures dry out and die.

#### 3.2.2. Marisma de Hinojos

**Figure 8:**
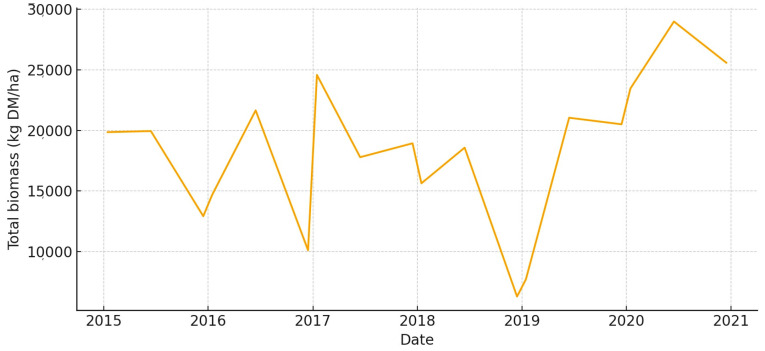
Total aboveground biomass (kg DM/ha) in *Marisma de Hinojos* (2015–2020).

**Figure 9:**
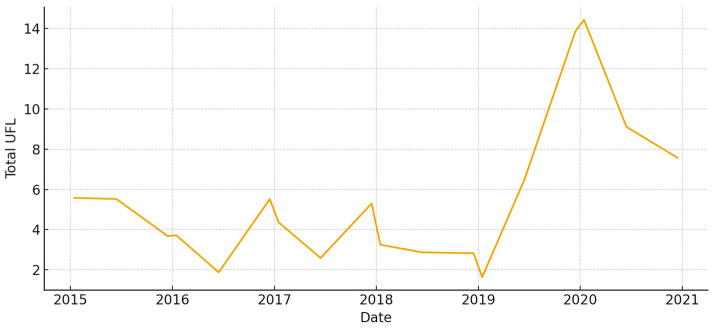
Total forage energy (UFL) in *Marisma de Hinojos* (2015–2020).

**Figure 10:**
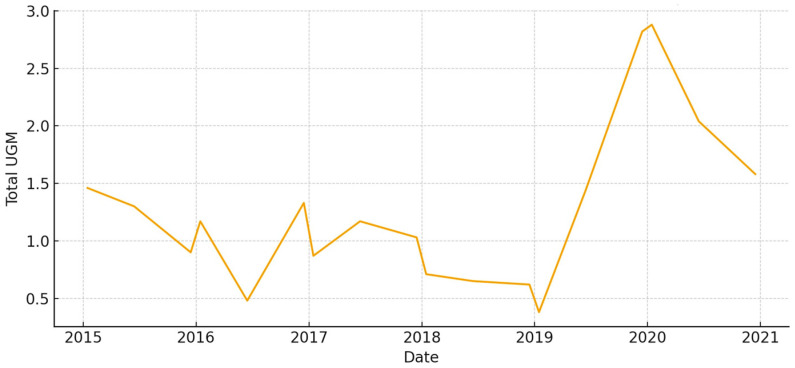
Total grazing pressure (UGM) in *Marisma de Hinojos* (2015–2020).

This large, low-lying unit has a lot of biomass in wet years, especially in the spring (Figure 8). But strong summer contractions happen when long hydroperiods make it harder to get to places. The total UFL is moderate during the main growth window, but it drops quickly once drawdown starts (Figure 9). Overall, Total UGM stays pretty low, but it goes up temporarily along the edges of the recession where grazeable edges form (Figure 10).

#### 3.2.3. Matochal

**Figure 11:**
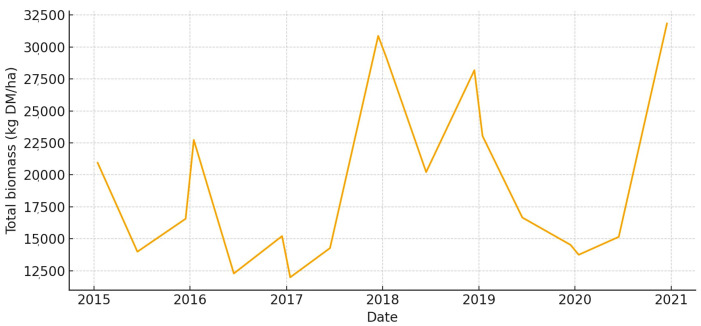
Total aboveground biomass (kg DM/ha) in *Matochal* (2015–2020).

**Figure 12:**
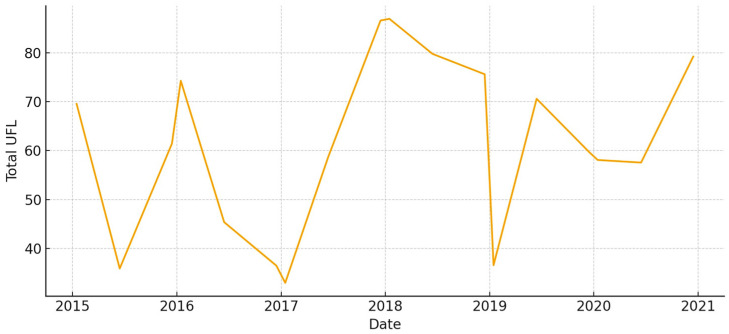
Total forage energy (UFL) in *Matochal* (2015–2020).

**Figure 13:**
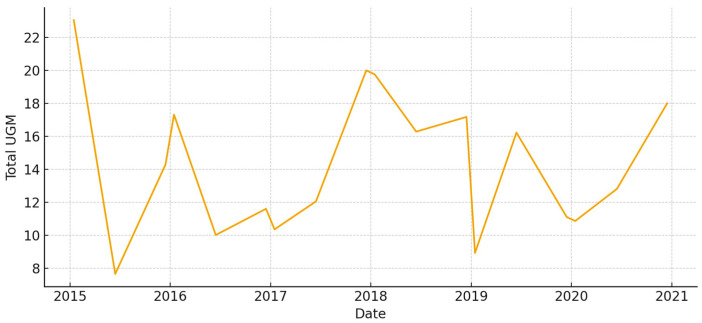
Total grazing pressure (UGM) in *Matochal* (2015–2020).

This unit is known for having a lot of variation from year to year. In years with long hydroperiods, total biomass peaks are delayed. In years with drier sequences, they appear earlier and shrink more quickly (Figure 11). When halophytic or lignified stands make up most of the communities at the end of the season, Total UFL stays relatively low (Figure 12). Total UGM shows these limits by only showing shallow peaks in the narrow window of maximum accessibility (Figure 13).

#### 3.2.4. La Vera y Sotos

**Figure 14:**
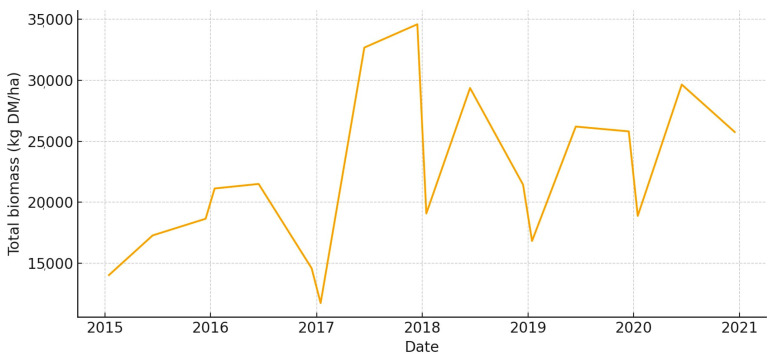
Total aboveground biomass (kg DM/ha) in *La Vera y Sotos* (2015–2020).

**Figure 15:**
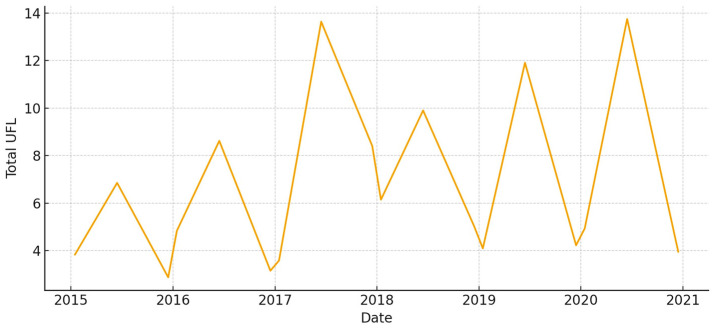
Total forage energy (UFL) in *La Vera y Sotos* (2015–2020).

**Figure 16:**
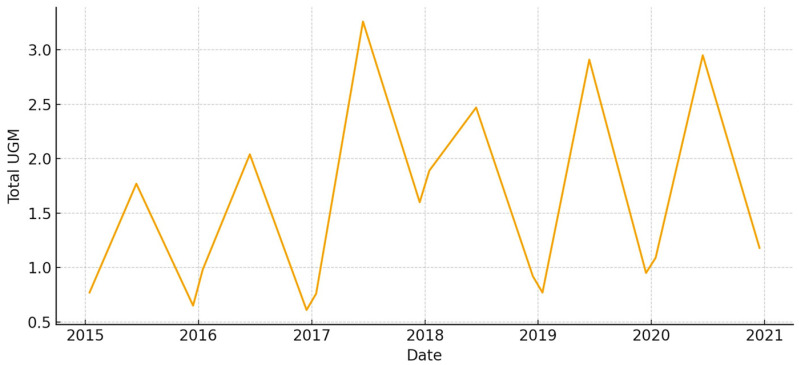
Total grazing pressure (UGM) in *La Vera y Sotos* (2015–2020).

This ecotonal belt always serves as a seasonal home. Total biomass stays at medium levels longer into the summer than it does in the low-marsh interiors (Figure 14). During the late-summer bottleneck, total UFL stays at relatively high levels (Figure 15). This is mostly because of the presence of perennial grasses and the early re-sprouting of annuals after the first rains. During dry spells, most of the total UGM is found along the vera (Figure 16), which shows how important microrelief and stable footing are for grazing.

#### 3.2.5. Las Nuevas y Marismillas

**Figure 17:**
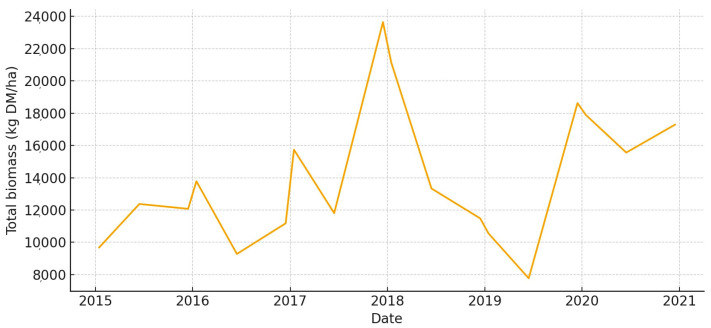
Total aboveground biomass (kg DM/ha) in *Las Nuevas y Marismillas* (2015–2020).

**Figure 18:**
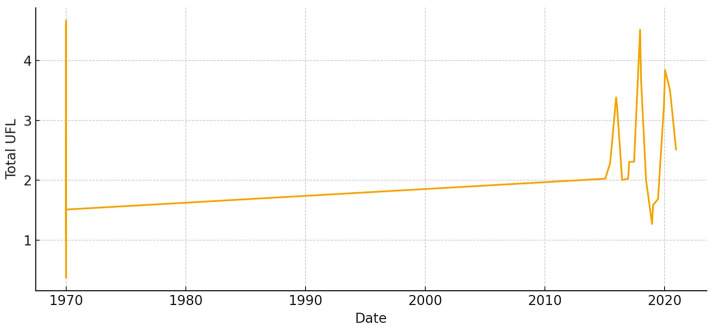
Total forage energy (UFL) in *Las Nuevas y Marismillas* (2015–2020).

**Figure 19:**
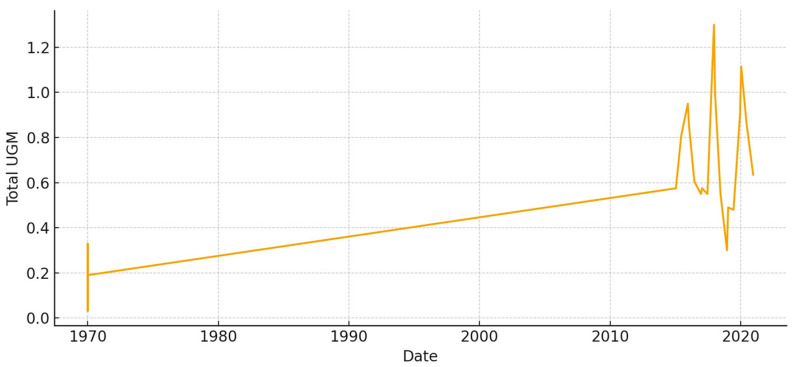
Total grazing pressure (UGM) in *Las Nuevas y Marismillas* (2015–2020).

When looked at as a whole, these marshlands that change with the water show strong seasonal responses. In wet years, total biomass increases a lot, but it drops a lot when the salinity and summer drought (Figure 17) happen. As the season goes on, the energetic return per unit mass goes down because total UFL drops earlier than biomass in saline depressions (Figure 18). Total UGM rises temporarily along recession margins (Figure 19), closely related to how easy it is to get to rather than how much biomass there is. The particular arrangement of Figure 18 and Figure 19 reflects the natural filling and drying cycle of the marsh in this specific study area. This hydrological pattern directly influences vegetation productivity and forage availability, resulting in the observed temporal distribution shown in figures.

### 3.3. Seasonal Windows of Suitability and Spatial Refugia

Looking at the total values across units shows that late winter to mid-spring is always the best time for grazing, with high total biomass and moderate-to-high total UFL. This window is especially good in *Marisma de Hinojos* and, in wetter years, *Matochal*. In contrast, late summer is the most limited time: total UFL values are very low across low-marsh interiors, even though there is still some biomass. *La Vera y Sotos* and some upland vetas serve as forage refuges during this bottleneck. They have higher total UFL and are easier to get to. *El Rincón* is a transitional unit that connects the dynamics of marshes and uplands and helps with seasonal shortages.

### 3.4. Sensor-Stack Performance and Uncertainty

The combined Sentinel-2 (L1C/L2A) and Landsat-8 (OLI/TIRS) datasets showed clear seasonal patterns in total biomass, total UFL, and total UGM across all units. Sentinel-2 made it easier to see ecotones and vera margins, which lowered the mixed-pixel effects in estimates of total forage quality and grazing pressure. Residual uncertainties arise from inundation and turbidity during peak flooding [34,35], pronounced salinity gradients, and changes in species composition, all of which can modify spectral–biophysical relationships, especially in late summer [35]. Even with these problems, the total trajectories at the unit level and the seasonal timing of suitability windows held up over time and across sensors.

## 4. Discussion

### 4.1. Seasonal and Interannual Patterns of Forage Dynamics

Our findings corroborate that seasonal cycles predominate forage dynamics in *Doñana*. During the cool-wet season, total biomass accumulation was always seen, but in the summer, when evapotranspiration was high and water was less available, it dropped quickly. The decrease in nutritional quality occurred before the loss of biomass, illustrating a well-documented quality–quantity decoupling in Mediterranean rangelands. This pattern is especially important for ungulates [37] like the *Marismeño* horse, which only eat natural grasses. In reality, the late summer bottleneck does not happen because there is not enough biomass; it happens because the forage that is available is not good enough to meet physiological needs.

Interannual variability also changes these seasonal cycles [39,42]. Wet years improved both biomass production and forage quality in the spring. However, long hydroperiods in large flats like *Marisma de Hinojos* made it harder to access the land, which reduced the amount of grazing that could be done. In contrast, dry years sped up the loss of biomass but made it easier to get to vera margins and upland patches, which served as grazing refuges. These different results show how important it is to think about both production and accessibility when figuring out carrying capacity.

### 4.2. The Role of Landscape Heterogeneity and Ecological Refugia

One of the most important things this study does is find ecotonal belts (*La Vera y Sotos*) and mixed marsh–upland mosaics (*El Rincón*) as important parts of the grazing landscape. During the summer, these units consistently had higher total UFL, which made them safe havens when the marshes inside became too unhealthy or too dry. These findings highlight the ecological significance of heterogeneity: varied soil types, microrelief, and hydrological gradients collectively mitigate seasonal shortages [34,35], supporting livestock and alleviating grazing pressure concentration.

From a management point of view, it is very important to protect and improve these refugia. These ecotones do more than just support livestock; they also support a lot of different kinds of plants and animals, including migratory birds and amphibians. So, managing horse grazing in a way that protects ecotones can help protect both cultural heritage and biodiversity.

### 4.3. Grazing Pressure Dynamics and Carrying Capacity Implications

Using summed UGM values as a proxy for grazing pressure helped us see how herds moved around in response to changes in resource availability. The results showed that there was always a delay between forage production and grazing pressure. This was due to both the animals’ ability to move around and the time it took for the marsh to become accessible. It is important to note that grazing peaks in late summer were mostly in ecotones, which raised concerns about overgrazing in certain areas. If there are too many animals in these refugia, the soil could become compacted, the plants could die, and biodiversity could be affected in many ways.

This shows how hard it is to take care of an endangered breed when they are free to roam. The *Marismeño* horse cannot be easily rotated or kept in a small space like livestock systems that are heavily managed. So, adaptive management needs to focus on tools that can dynamically assess carrying capacity and warn of problems with forage suitability before they happen. The remote sensing workflow created here is a step toward these kinds of flexible frameworks.

### 4.4. Methodological Contributions and Limitations

The methodological framework presented here expands upon earlier remote sensing applications for rangeland monitoring by integrating spectral, temporal, and ecological dimensions at a higher level of spatial precision. Unlike traditional models based on MODIS or single-index NDVI data [28], our approach harmonizes Sentinel-2 and Landsat 8 imagery to simultaneously estimate biomass, forage quality, and grazing pressure at a 10–30 m resolution [40]. This refinement enables the detection of short-term ecological responses to hydroperiod shifts and salinity stress, factors that strongly influence vegetation dynamics in *Doñana*. Moreover, by combining multi-year image series with Python-based modeling, our approach transforms remote sensing from a descriptive tool into a decision support system capable of informing adaptive management strategies for free-ranging livestock in complex Mediterranean wetland environments.

On the other hand, the amalgamation of Sentinel-2 and Landsat 8 datasets constituted a significant advantage [40]. Sentinel-2’s higher spatial resolution (10 m) made it easier to find narrow vera margins and ecotonal strips, which are important for functionality but are often not clear in coarser images. Landsat 8, on the other hand, gave us a longer time series and thermal data, which made it possible to compare data from different years. The harmonization process (cubic convolution rescaling of TIRS bands, nearest-neighbor resampling, and co-registration) made sure that all the sensors worked together, which is an important step for temporal analysis [31,32,33,34].

Still, there are some limits that still exist. Cloud cover in the winter made it hard to obtain images, so temporal interpolation was needed. During flooding, high turbidity and water reflectance sometimes made vegetation indices hard to read. Also, halophytic communities had spectral signatures that were hard to tell apart from senescent biomass. These constraints indicate that the amalgamation of hyperspectral datasets or both high-resolution satellite and UAV-based observations could enhance forage quality estimations [22,24,28].

### 4.5. Conservation and Cultural Dimensions

The *Marismeño* horse is not just a genetic resource; it is also a cultural symbol of Andalusian identity [41]. The *Saca de las Yeguas* and other events show how closely connected this breed is to local communities. Our research helps protect this cultural heritage by measuring the ecological limits of its free-range system. The study significantly demonstrates the integration of contemporary remote sensing with traditional methodologies, thereby reconciling innovation with heritage conservation.

At the same time, conservation goals must take into account the trade-offs between cultural livestock use and biodiversity goals. For example, too much grazing during dry years could hurt the needs of marsh birds that are in danger. This method gives managers, conservationists, and local stakeholders clear numbers on carrying capacity, which makes it easier for them to talk about these trade-offs.

### 4.6. Future Perspectives Under Climate Change

In accordance with [39], projected climate change presents further challenges for *Doñana*. More frequent droughts, less aquifer recharge, and changes in the timing of rainfall may make flooding shorter, saltier, and less productive [34,35]. In these conditions, the late-summer bottleneck may last longer and be worse, putting more stress on the *Marismeño* horse population. Our results indicate that refugia like *La Vera y Sotos* will become increasingly vital in the coming decades.

To prepare for these changes, scenario-based modeling should combine climate forecasts, hydrological models, and remote sensing data. By connecting these scenarios to dynamic carrying capacity models, managers could look at different climate futures and see how their plans would work. Additionally, combining forage monitoring with horse movement data (e.g., GPS tracking) would give direct information about how herds react to changes in resource landscapes.

### 4.7. Implications for Sustainability and the Sustainable Development Goals (SDGs)

This study also fit with the global sustainability goals set by the UN 2030 Agenda (https://sdgs.un.org/2030agenda, accessed on 2 December 2025) [43]. The work directly supports SDG 15 (Life on Land) by measuring the carrying capacity of *Doñana*’s marshlands. SDG 15 focuses on protecting biodiversity and using terrestrial ecosystems in a way that is sustainable [44]. The *Marismeño* horse is an endangered breed of livestock and a cultural symbol. It represents the connection between protecting agrobiodiversity and preserving heritage. The remote sensing workflow also helps with SDG 6 (Clean Water and Sanitation) by showing how important water availability, hydroperiod length, and salinity gradients are to forage dynamics. This shows how important sustainable hydrological management is for both ecological and pastoral functions. Lastly, this framework helps SDG 13 (Climate Action) by giving us tools to keep an eye on how climate change affects forage resources and to come up with ways to adapt that lower the risks to livestock, ecosystems, and cultural traditions. Adding remote sensing to management not only makes *Doñana*’s ecosystems more resilient, but it also shows a way to reach sustainability goals in other rangeland systems around the world that are vulnerable to climate change.

### 4.8. Broader Applicability

This study centers on *Doñana*, yet its methodological framework possesses wider applicability. Many free-ranging livestock systems around the world face the same problems: big changes in the seasons, climate-driven forage bottlenecks, and the need to find a balance between conservation and cultural traditions. The workflow shown here, which combines remote sensing, GIS, and machine learning, can be used in other protected areas where there is not enough data or management problems make it hard to do regular monitoring. Remote sensing can help make better decisions about managing rangelands around the world by giving objective, spatially explicit measures of how much forage is available and how much land can support it.

### 4.9. Synthesis and Key Contributions

In summary, the discussion brings up a few important points:

1. Remote sensing showed a clear separation between biomass and forage quality, especially in late summer. This shows how easy it is to overestimate carrying capacity.
2. Ecotones and mixed uplands have become important refuges that help with seasonal shortages and should be given top priority in management.
3. Interannual variability caused by the length of the hydroperiod showed that we need to monitor things over a long period of time and be able to change our plans as needed.
4. The harmonization of multi-sensor datasets was effective for multi-year ecological assessments, although some uncertainties remained.
5. To protect the *Marismeño* horse, we need to look at it from cultural, ecological, and climate points of view. Remote sensing is a useful tool for making decisions about this.

## 5. Conclusions

This study shows the efficacy of satellite remote sensing as a robust, non-invasive, and scalable instrument for evaluating forage dynamics and rangeland carrying capacity in Mediterranean wetland ecosystems. We used multi-sensor images from Sentinel-2 (L1C/L2A) and Landsat 8 (OLI/TIRS) along with spatially explicit grazing unit boundaries to give a detailed six-year (2015–2020) look at biomass production, forage quality, and grazing pressure for the *Marismeño* horse in *Doñana* National Park.

The findings indicate a markedly seasonal system governed by hydrological and climatic limitations. During the cool, wet months, biomass builds up, but it drops sharply in the summer. The nutritive value (UFL) drops even earlier when salinity and senescence rise. This separation between quantity and quality shows how limited traditional biomass-based assessments are and how important spectral indicators that measure both vegetation vigor and water content are. Interannual variability was pronounced, reflecting fluctuations in rainfall and hydroperiod length, which in turn modulate forage availability and accessibility across the park’s heterogeneous landscape.

Ecotonal and upland mosaics, especially *La Vera y Sotos* and *El Rincón*, always worked as seasonal refuges by keeping intermediate biomass and higher nutritional quality during the late-summer bottleneck. On the other hand, large low-marsh units like *Marisma de Hinojos* and *Las Nuevas y Marismillas* had high productivity in the spring but were very hard to get to when they were flooded for a long time. These differences in space show how important it is for the environment to have a variety of landscapes to help with seasonal shortages and guide the adaptive management of free-ranging herds.

The study methodologically confirmed the efficacy of integrating Sentinel-2 and Landsat 8 archives for monitoring fine-scale ecological processes over extended temporal spans. The combination of the GNDVI and NDMI indices was able to accurately measure biomass production and how halophytic plants reacted to changes in salinity. Even though there are still some questions about turbidity, cloud cover, and spectral ambiguity in mixed halophyte-grass assemblages, the time series that came out of it was strong enough to find trends and strange things in forage dynamics.

From a conservation standpoint, the results offer quantitative evidence that the sustainability of the *Marismeño* horse relies on adaptive management strategies responsive to spatio-temporal fluctuations in resource availability. To keep herd sizes healthy, grazing intensity needs to match real-time assessments of carrying capacity while also protecting important ecotonal refugia. The methodology established herein provides a decision support framework that can guide future management strategies for *Doñana* and analogous protected landscapes where traditional pastoralism coexists with significant ecological value.

In the future, combining this remote sensing method with field data on things like soil moisture, vegetation composition, and animal movement (like GPS tracking) would make it possible to model resource use and ecosystem feedback in a more complete way. This kind of integration could also make early warning systems for stress caused by drought better, which would help protect both livestock and biodiversity. The workflow can also be used in other Mediterranean and semi-arid systems that have similar problems with not having enough water, having habitats broken up, and having the weather change.

This research contributes to the attainment of the Sustainable Development Goals (SDGs 6, 13, and 15) by enhancing understanding of the relationships among water management, climate resilience, and biodiversity conservation. It shows how science-based, data-driven methods can connect conservation and cultural heritage by using Earth observation technologies. This makes sure that iconic breeds like the *Marismeño* horse can live on in healthy ecosystems.

## Data Availability

The original contributions presented in the study are included in the article; further inquiries can be directed to the corresponding author.
